# Supplementary material for: Differences in Penile Hemodynamic Profiles in Patients with Erectile Dysfunction and Anxiety
Source: J Clin Med. 2021 Jan 21;10(3):402. doi: 10.3390/jcm10030402 (PMC7864480; doi:10.3390/jcm10030402)
Supplement: Supplementary file 1 [file jcm-10-00402-s001.pdf]

Table S1. The 7-items of the Generalized Anxiety Disorder (GAD-7) score.

GAD-7 Questionnaire				
**Over the Last 2 Weeks, How Often Have You Been Bothered by the Following Problems?**				
	Not at all	Several days	More than half the days	Nearly every day
1. Feeling nervous, anxious or on edge	0	1	2	3
2. Not being able to stop or control worrying	0	1	2	3
3. Worrying too much about different things	0	1	2	3
4. Trouble relaxing	0	1	2	3
5. Being so restless that it is hard to sit still	0	1	2	3
6. Becoming easily annoyed or irritable	0	1	2	3
7. Feeling afraid as if something awful might happening	0	1	2	3

Table S2. Peak systolic velocity of groups at each time-point assessment.

Dependent Measure		Comparison	Difference of the Mean	SE	*p*	95% CI
PSV (time 0 min)	Group 1	Group 2	0.50	0.65	0.45	−0.80–1.80
		Group 3	0.25	0.65	0.70	−1.04–1.55
		Group 4	0.70	0.65	0.29	−0.60–2.00
	Group 2	Group 1	−0.50	0.65	0.45	−1.80–0.80
		Group 3	−0.25	0.65	0.70	−1.55–1.05
		Group 4	0.20	0.65	0.76	−1.10–1.50
	Group 3	Group 1	−0.25	0.65	0.70	−1.55–1.05
		Group 2	0.25	0.65	0.70	−1.05–1.55
		Group 4	0.45	0.65	0.49	−0.85–1.75
	Group 4	Group 1	−0.70	0.65	0.29	−2.00–0.60
		Group 2	−0.20	0.65	0.76	−1.50–1.10
		Group 3	−0.45	0.65	0.49	−1.75–0.85
PSV (time 5 min)	Group 1	Group 2	9.65	2.06	<0.01	5.54–13.76
		Group 3	23.55	2.06	<0.01	19.44–27.66
		Group 4	45.10	2.06	<0.01	40.99–49.21
	Group 2	Group 1	−9.65	2.06	<0.01	−13.76–−5.54
		Group 3	13.90	2.06	<0.01	9.79–18.01
		Group 4	35.45	2.06	<0.01	31.34–39.56
	Group 3	Group 1	−23.55	2.06	<0.01	−27.66–−19.44
		Group 2	−13.90	2.06	<0.01	−18.01–−9.79
		Group 4	21.55	2.06	<0.01	17.44–25.66
	Group 4	Group 1	−45.10	2.06	<0.01	−49.21–−40.99
		Group 2	−35.45	2.06	<0.01	−39.56–−31.34
		Group 3	−21.55	2.06	<0.01	−25.66–−17.44
PSV (time 10 min)	Group 1	Group 2	8.90	2.05	<0.01	4.82–12.98
		Group 3	22.80	2.05	<0.01	18.72–26.88
		Group 4	43.05	2.05	<0.01	38.97–47.13
	Group 2	Group 1	−8.90	2.05	<0.01	−12.98–−4.82
		Group 3	13.90	2.05	<0.01	9.82–17.98
		Group 4	34.15	2.05	<0.01	30.07–38.23
	Group 3	Group 1	−22.80	2.05	<0.01	−26.88–−18.72
		Group 2	−13.90	2.05	<0.01	−17.98–−9.82
		Group 4	20.25	2.05	<0.01	16.17–24.33
	Group 4	Group 1	−43.05	2.05	<0.01	−47.13–38.97
		Group 2	−34.15	2.05	<0.01	−38.23–−30.07
		Group 3	−20.25	2.05	<0.01	−24.33–−16.17

PSV (time 15 min)	Group 1	Group 2	8.60	2.00	<0.01	4.62–12.58
		Group 3	22.20	2.00	<0.01	18.21–26.18
		Group 4	34.10	2.00	<0.01	30.12–38.08
	Group 2	Group 1	−8.60	2.00	<0.01	−12.58–−4.62
		Group 3	13.60	2.00	<0.01	9.62–17.58
		Group 4	25.50	2.00	<0.01	21.51–29.48
	Group 3	Group 1	−22.20	2.00	<0.01	−26.18–18.22
		Group 2	−13.60	2.00	<0.01	−17.58–−9.62
		Group 4	11.90	2.00	<0.01	7.92–15.88
	Group 4	Group 1	−34.10	2.00	<0.01	−38.08–−30.12
		Group 2	−25.50	2.00	<0.01	−29.48–−21.52
		Group 3	−11.90	2.00	<0.01	−15.88–−7.92
PSV (time 20 min)	Group 1	Group 2	10.15	2.16	<0.01	5.86–14.44
		Group 3	22.50	2.16	<0.01	18.20–26.79
		Group 4	20.40	2.16	<0.01	16.11–24.69
	Group 2	Group 1	−10.15	2.16	<0.01	−14.44–5.86
		Group 3	12.35	2.16	<0.01	8.06–16.64
		Group 4	10.25	2.16	<0.01	5.96–14.54
	Group 3	Group 1	−22.50	2.16	<0.01	−26.79–−18.21
		Group 2	−12.35	2.16	<0.01	−16.64–−8.06
		Group 4	−2.10	2.16	0.33	−6.39–2.19
	Group 4	Group 1	−20.40	2.16	<0.01	−24.69–−16.11
		Group 2	−10.25	2.16	<0.01	−14.54–−5.96
		Group 3	2.1	2.16	0.33	−2.19–6.39

Data are analyzed using the multivariate analysis of variance (MANOVA), with post-hoc LSD analysis. PSV = peak systolic velocity; SE = standard error.

Table S3. Effect size measure of peak systolic velocity.

	Test	Value	F	Effect df	Error df	*p*	Partial Eta Squared	Non Centrality
**Intercept**	Pillai	0.99	1008.02	5.00	72.00	<0.01	0.99	5040.08
	Wilk	0.14	1008.02	5.00	72.00	<0.01	0.99	5040.08
	Hotelling	70.00	1008.02	5.00	72.00	<0.01	0.99	5040.08
	Roi	70.00	1008.02	5.00	72.00	<0.01	0.99	5040.08
**GAD-7**	Pillai	1.51	15.01	15.00	222.00	<0.01	0.50	225.15
	Wilk	0.03	33.21	15.00	199.16	<0.01	0.68	431.73
	Hotelling	14.31	67.43	15.00	212.00	<0.01	0.83	1011.37
	Roi	13.12	194.15	5.00	74.00	<0.01	0.93	970.73

Table S4. End-diastolic velocity of groups at each time-point assessment.

Dependent Measure		Comparison	Difference of the Mean	SE	*p*	95% CI
**EDV (time 5 min)**	Group 1	Group 2	−0.85	0.29	<0.01	−1.42–−0.28
		Group 3	−1.25	0.29	<0.01	−1.82–−0.68
		Group 4	−2.75	0.29	<0.01	−3.32–−2.18
	Group 2	Group 1	0.85	0.29	<0.01	0.28–1.42
		Group 3	−0.40	0.29	0.17	−0.97–0.17
		Group 4	−1.9	0.29	<0.01	−2.47–−1.33
	Group 3	Group 1	1.25	0.29	<0.01	0.68–1.82
		Group 2	0.40	0.29	0.17	−0.17–0.97
		Group 4	−1.50	0.29	<0.01	−2.07–−0.93
	Group 4	Group 1	2.75	0.29	<0.01	2.18–3.32
		Group 2	1.90	0.29	<0.01	1.33–2.47
		Group 3	1.50	0.29	<0.01	0.93–2.07
**EDV (time 10 min)**	Group 1	Group 2	−0.50	0.30	0.10	−1.11–0.11
		Group 3	−1.30	0.30	<0.01	−1.91–−0.69
		Group 4	−1.85	0.30	<0.01	−2.46–−1.24

	Group 2	Group 1	0.50	0.30	0.10	−0.11–1.11
		Group 3	−0.80	0.30	0.01	−1.41–−0.19
		Group 4	−1.35	0.30	<0.01	−1.96–0.74
	Group 3	Group 1	1.30	0.30	<0.01	0.69–1.91
		Group 2	0.80	0.30	0.01	0.19–1.41
		Group 4	−0.55	0.30	0.07	−1.16–0.06
	Group 4	Group 1	1.85	0.30	<0.01	1.24–2.46
		Group 2	1.35	0.30	<0.01	0.74–1.96
		Group 3	0.55	0.30	0.07	−0.06–1.16
**EDV (time 15 min)**	Group 1	Group 2	−0.55	0.25	0.03	−1.06–−0.04
		Group 3	−1.35	0.25	<0.01	−1.86–−0.84
		Group 4	−1.00	0.25	<0.01	−1.51–−0.49
	Group 2	Group 1	0.55	0.25	0.03	0.04–1.06
		Group 3	−0.80	0.25	<0.01	−1.31–−0.29
		Group 4	−0.45	0.25	0.08	−0.96–0.06
	Group 3	Group 1	1.35	0.25	<0.01	0.84–1.86
		Group 2	0.80	0.25	<0.01	0.29–1.31
		Group 4	0.35	0.25	0.17	−0.16–0.86
	Group 4	Group 1	1.00	0.25	<0.01	0.49–1.51
		Group 2	0.45	0.25	0.08	−0.06–0.96
		Group 3	−0.35	0.25	0.17	−0.86–0.16
**EDV (time 20 min)**	Group 1	Group 2	−0.90	0.24	<0.01	−1.37–−0.43
		Group 3	−1.60	0.24	<0.01	−2.07–−1.13
		Group 4	−0.50	0.24	0.04	−0.97–−0.03
	Group 2	Group 1	0.90	0.24	<0.01	0.43–1.37
		Group 3	−0.70	0.24	<0.01	−1.17–−0.23
		Group 4	0.40	0.24	0.10	−0.07–0.87
	Group 3	Group 1	1.60	0.24	<0.01	1.13–2.07
		Group 2	0.70	0.24	<0.01	0.23–1.17
		Group 4	1.10	0.24	<0.01	0.63–1.57
	Group 4	Group 1	0.50	0.24	0.04	0.03–0.97
		Group 2	−0.40	0.24	0.10	−0.87–0.07
		Group 3	−1.10	0.24	<0.01	−1.57–−0.63

Data are analyzed using the multivariate analysis of variance (MANOVA), with post-hoc LSD analysis. EDV = end-diastolic velocity; SE = standard error.

Table S5. Effect size measure of end-diastolic velocity.

	Test	Value	F	Effect df	Error df	*p*	Partial Eta Squared	Non Centrality
**Intercept**	Pillai	0.64	25.99	5.00	72.00	<0.01	0.64	129.93
	Wilk	0.36	25.99	5.00	72.00	<0.01	0.64	129.93
	Hotelling	1.81	25.99	5.00	72.00	<0.01	0.64	129.93
	Roi	1.81	25.99	5.00	72.00	<0.01	0.64	129.93
**GAD-7**	Pillai	1.15	9.18	15.00	222.00	<0.01	0.38	137.71
	Wilk	0.19	11.08	15.00	199.16	<0.01	0.43	148.95
	Hotelling	2.68	12.61	15.00	212.00	<0.01	0.47	189.11
	Roi	1.91	28.21	5.00	74.00	<0.01	0.66	141.06

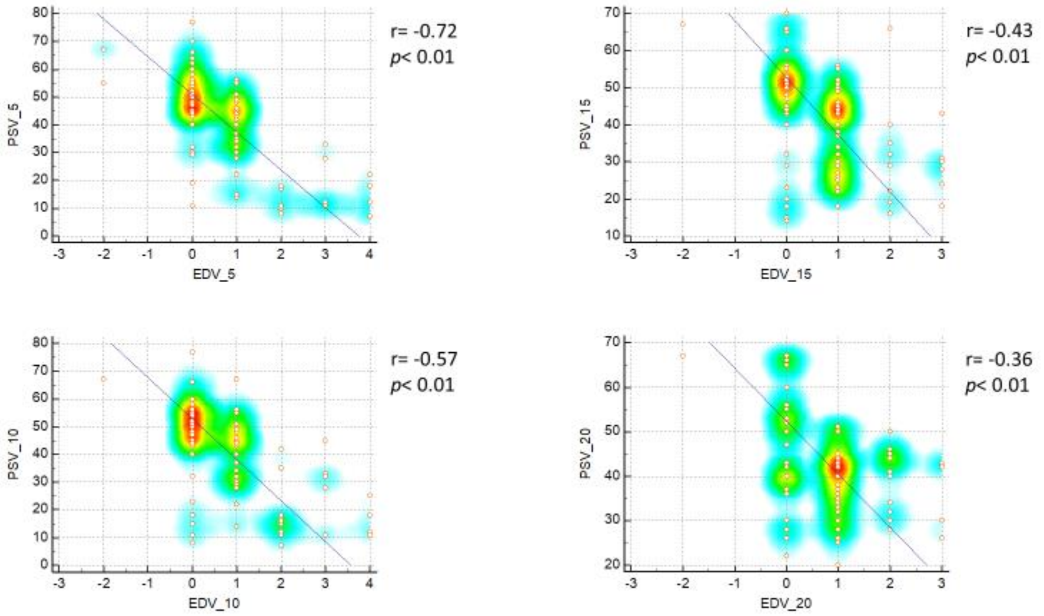

Figure S1. Correlation analysis between peak systolic velocity (PSV) and end-diastolic velocity (EDV), at each time-point of assessment. The correlation analysis was evaluated by calculating the Pearson coefficient (r).
